# A Novel Intelligent System for Dynamic Observation of Cotton Verticillium Wilt

**DOI:** 10.34133/plantphenomics.0013

**Published:** 2023-01-10

**Authors:** Chenglong Huang, Zhongfu Zhang, Xiaojun Zhang, Li Jiang, Xiangdong Hua, Junli Ye, Wanneng Yang, Peng Song, Longfu Zhu

**Affiliations:** ^1^ College of Engineering, Huazhong Agricultural University, Wuhan 430070, PR China.; ^2^ College of Plant Science & Technology, Huazhong Agricultural University, Wuhan 430070, PR China.; ^3^ National Key Laboratory of Crop Genetic Improvement, National Center of Plant Gene Research (Wuhan), Huazhong Agricultural University, Wuhan 430070, PR China.

## Abstract

Verticillium wilt is one of the most critical cotton diseases, which is widely distributed in cotton-producing countries. However, the conventional method of verticillium wilt investigation is still manual, which has the disadvantages of subjectivity and low efficiency. In this research, an intelligent vision-based system was proposed to dynamically observe cotton verticillium wilt with high accuracy and high throughput. Firstly, a 3-coordinate motion platform was designed with the movement range 6,100 mm × 950 mm × 500 mm, and a specific control unit was adopted to achieve accurate movement and automatic imaging. Secondly, the verticillium wilt recognition was established based on 6 deep learning models, in which the VarifocalNet (VFNet) model had the best performance with a mean average precision (*mAP*) of 0.932. Meanwhile, deformable convolution, deformable region of interest pooling, and soft non-maximum suppression optimization methods were adopted to improve VFNet, and the *mAP* of the VFNet-Improved model improved by 1.8%. The precision–recall curves showed that VFNet-Improved was superior to VFNet for each category and had a better improvement effect on the ill leaf category than fine leaf. The regression results showed that the system measurement based on VFNet-Improved achieved high consistency with manual measurements. Finally, the user software was designed based on VFNet-Improved, and the dynamic observation results proved that this system was able to accurately investigate cotton verticillium wilt and quantify the prevalence rate of different resistant varieties. In conclusion, this study has demonstrated a novel intelligent system for the dynamic observation of cotton verticillium wilt on the seedbed, which provides a feasible and effective tool for cotton breeding and disease resistance research.

## Introduction

Cotton is one of the most important economic crops widely planted all over the world [1]. Meanwhile, in the world’s major cotton areas, verticillium wilt is regarded to be the main disease of cotton production [2,3], because of its extensive transmission route, serious harm, and complex infection mechanism [4]. The cotton verticillium wilt is generally infected by soil fungi including *Verticillium dahliae* and *Verticillium albo-atrum*, which would cause the leaves to wilt, fade, and fall off [5,6]. More seriously, the growth and development of cotton would slow down or even wither, which would finally result in the decline of cotton quality and yield [7]. Therefore, the real-time and accurate evaluation of cotton verticillium wilt is crucial to the cotton disease resistance research.

At present, the evaluation methods of cotton verticillium wilt mainly include manual investigation, remote sensing observation, and hyperspectral measurement [8,9]. Manual investigation mainly depends on visual observation and personal experience, which may result in low efficiency and poor consistency. Besides, due to the cotton verticillium wilt invasion, the cell structure, water content, and nitrogen content of crop leaves would change, which would also result in the variation of corresponding spectral information [10,11]. Jin *et al.* [9] once analyzed the hyperspectral reflectance data of cotton leaves with different disease degrees to establish the characterization model of verticillium wilt by machine learning algorithms including discriminant analysis, back propagation neural network, and support vector machine. However, this method has the disadvantages of high cost, low efficiency, and poor flexibility. Thus, it would have great practicality value to develop a cotton verticillium wilt observation platform, in which the reliable and adaptable algorithm for cotton verticillium wilt recognition should be developed [12,13].

The plant phenotyping platform is a large-scale research facility that integrates a transport unit, an image collection unit, and image analysis and storage unit, which could extract crop phenotypic traits with high throughput and high accuracy [14]. There has been an increase in research on plant phenotyping platform these past few years both locally and globally. The rice phenotyping system developed by Huazhong Agriculture University was able to extract the rice plant height, biomass, tiller, and panicle information [15]. The Australian plant phenotyping accelerator can capture the images with throughput of 2,400 plants [16]. The above plant phenotyping platforms are based on the “plant to sensor” mode. However, in some settings, plants cannot be moved to sensors; therefore, the “sensor to plant” platforms are developed. The Crop3D designed by the Chinese Academy Institution of Sciences has installed lidar, high-resolution visible light and hyperspectral cameras, which could be used to extract plant traits in multiscale and multigrowth periods [17]. The phenotyping platform in UK Lausanne station was equipped with multiple sensors, which has been applied to wheat and other crops with a field coverage of 10 × 120 m [18]. The vehicle phenotyping platform designed by Barker et al. [19] could obtain the field crop information with high flexibility and efficiency. Texas et al. have adopted unmanned aerial vehicles to evaluate the cotton canopy density and pest-damaged areas. In conclusion, it will be of great significance to develop a cotton seedling phenotyping platform for verticillium wilt observation [20].

After establishing the imaging platform, it is crucial to analyze the images for plant traits. In recent years, with the rapid development of deep learning and computer vision technology, a large number of excellent object detection algorithms have emerged. According to whether candidate regions are proposed, the detection algorithms can be divided into 1-stage and 2-stage algorithms. The 1-stage object detection algorithm such as Single Shot MultiBox Detector (SSD) [21], RetinaNet [22], VarifocalNet (VFNet) [23], and You Only Look One-level Feature (YOLOF) [24] can predict the bounding boxes (bboxes) directly. Multistage object detection algorithms including Faster Region-based Convolutional Neural Networks (R-CNN) [25] and Cascade R-CNN [26] firstly extract anchors from feature images and then make secondary corrections to obtain detection results. The present research shows that these deep learning algorithms are able to provide high-precision and reliable image analysis methods, which have been widely applied in the identification of diseases, pests, and weeds [12,27–29] and fruit detection and plant counting [30,31]. Lu et al. [32] have proposed a field wheat disease diagnosis system based on weak supervision and deep learning architecture. The weed identification system proposed by Espejo-Garcia et al. [33] was able to identify and locate early weeds in the field, so as to reduce the utilization rate of pesticides. Chen et al. [30] have realized online detection and tracking of defective citrus based on deep sort tracker. Ghosal et al. [34] published a weakly supervised deep learning framework for sorghum ears counting by unmanned aerial vehicles. Velumani et al. [35] adopted a Faster-RCNN detection model to estimate the maize plant density. Liu et al. [36] applied the dynamic color transform networks for the wheat head detection based on YOLOV4. In general, image processing algorithm based on deep learning has been proven to be effective in the field of agriculture and also provided a feasible and powerful method for this study.

In this research, an automatic and intelligent system on the seedbed has been designed, which could dynamically observe the cotton verticillium wilt and obtain quantitative pathological data with high throughput and high accuracy, which is of great significance for cotton breeding and disease resistance research. The system was realized by developing a specialized hardware and software platform including system control software and cotton verticillium wilt recognition software based on VFNet-Improved, which would definitely improve the accuracy and automation in verticillium wilt investigation, and it would provide an efficient and reliable tool for cotton research.

## Materials and Methods

**Fig. 1.**
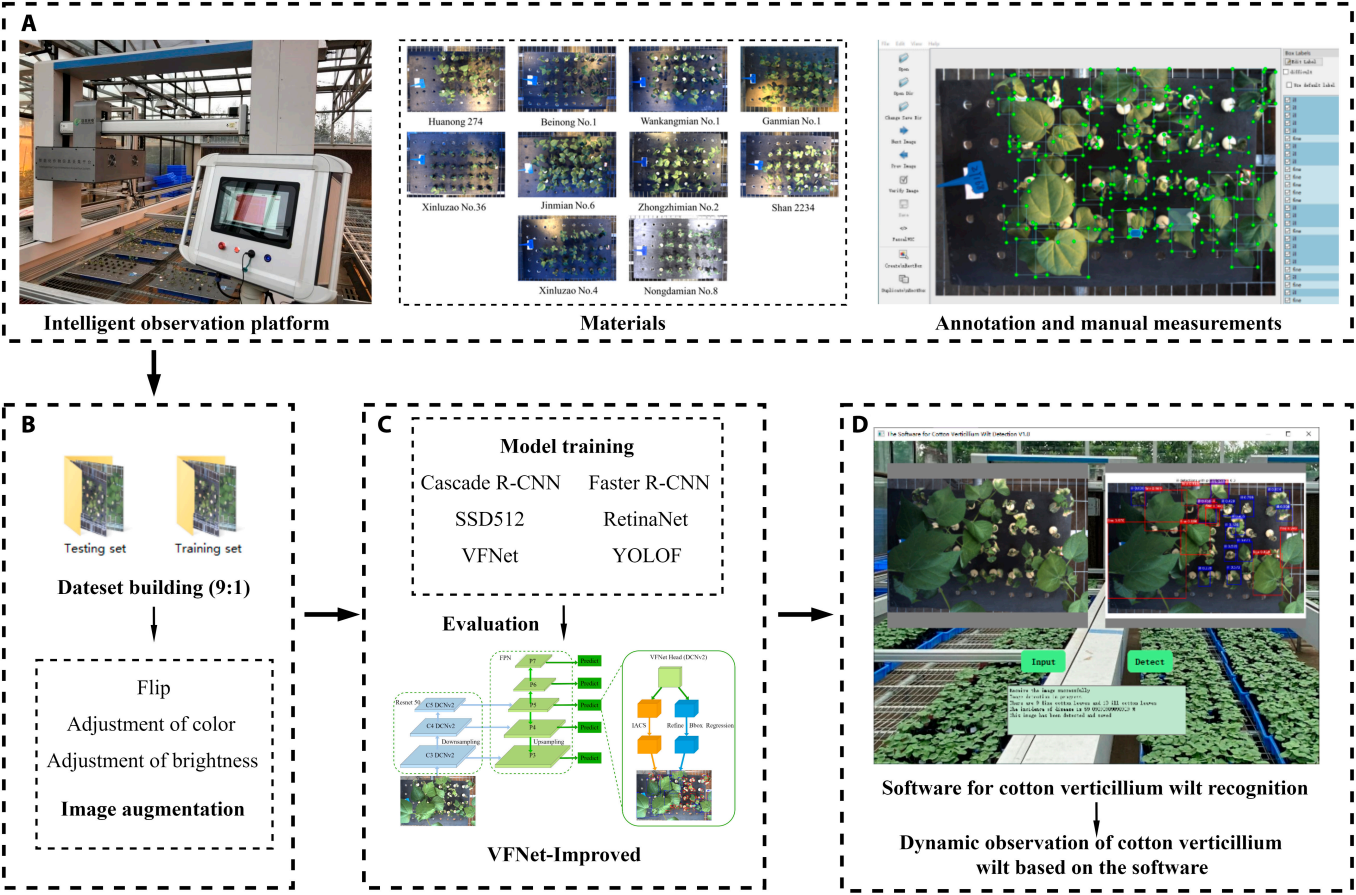
Technique framework for the cotton verticillium wilt observation system.

The overall technique framework for the cotton verticillium wilt observation system is shown in Fig. 1, which consists of the system design (Fig. 1A), experiment design (Fig. 1B), model optimization (Fig. 1C), and user software design (Fig. 1D) for cotton verticillium wilt investigation. The detailed information of each module is provided in the following sections.

### System design

**Fig. 2.**
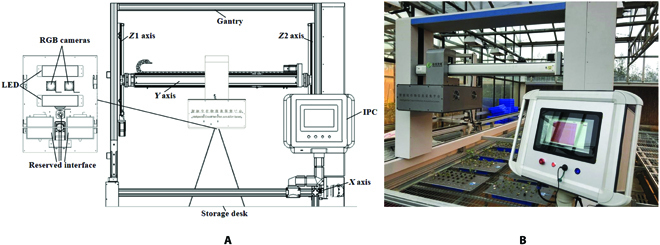
The system design (A) and application (B) of the intelligent observation platform for cotton verticillium wilt.

The system design and application of the intelligent observation platform for cotton verticillium wilt are shown in Fig. 2A and B, respectively. The system mainly consisted of the 3-coordinate motion unit, image acquisition unit, and control unit. The main structure of the platform was made by aluminum alloy materials, which were installed on the seedbed, and the size is 7,000 × 2,000 × 1,950 mm. A 3-axis motion unit was adopted in the system, which was driven by a stepping motor and an accurate motion control card (ECI2000, China Zmotion), and the motion distance of each axis was 6,100 mm for the *X* axis, 950 mm for the *Y* axis, and 500 mm for the *Z* axis. The RGB camera (MARS-1230-23U3C, China Daheng) was adopted as an image acquisition unit, which was installed in the *Y* axis. The pixel resolution was 4,096 × 3,000 pixels, while a 16-mm lens was applied to obtain the 530 mm × 388 mm visual field. The camera was connected to an industrial personal computer (IPC; ARK-3500, China YanHua) by a USB3.0 data interface, which could realize efficient and stable image transmission. Finally, the system was manufactured by GreenPheno Co., Ltd. The basic hardware parameters of the platform are shown in Table 1, which was able to achieve rapid and automatic acquisition of cotton images on the seedbed.

**Table 1.** Platform hardware parameters

Main structure	*X* axis	7,000 mm
	*Y* axis	2,000 mm
	*Z* axis	1,950 mm
Movement range	*X* axis	6,100 mm
	*Y* axis	950 mm
	*Z* axis	500 mm
Camera parameter	Resolution	4,096 × 3,000
	Pixel size	3.45 × 3.45 μm
	Core chip	Sony IMX304 CMOS
	Focal length	16 mm
	Interface	USB3.0

### System control and workflow

**Fig. 3.**
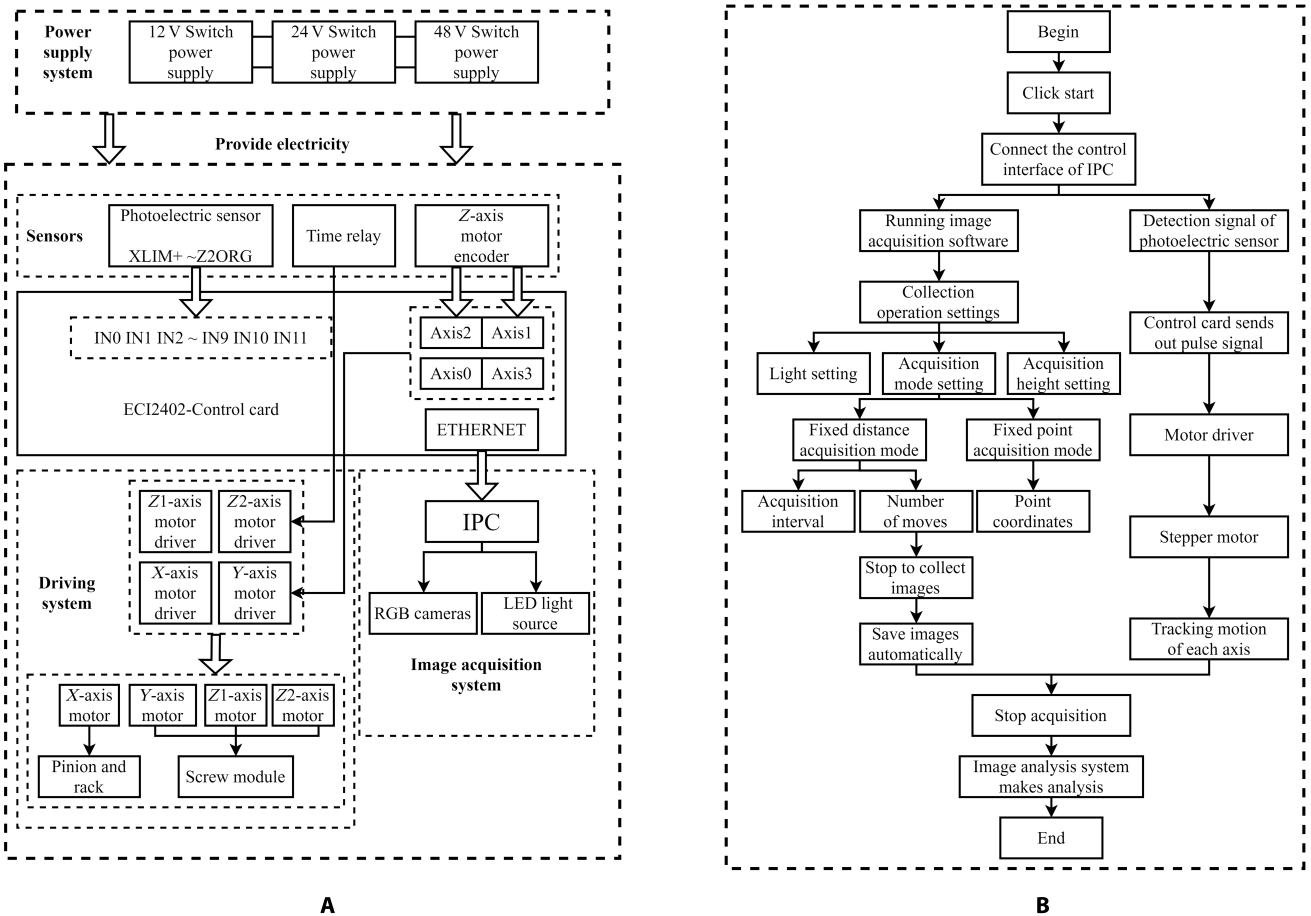
The system control (A) and workflow (B) of the intelligent cotton information acquisition platform.

The system control including axis motion and image acquisition was achieved by programming in the IPC and the motion control card, and the control diagram is shown in Fig. 3A. The motion control unit was able to perform independent of linkage motion for each axis with a predetermined speed and acceleration. Meanwhile, the position sensors for origin return, position feedback, and safety protection were applied to achieve a position accuracy of 0.01 mm. The motion control software was developed on the visual studio 2017 platform, and the user interface was designed by C# language. The dynamic link libraries provided by the ECI2000 card was used to control the 3-coordinate motion platform. With the high-precision motion control system, an automatic imaging method was carried out by a defined motion locus, which took only 4 min to automatically capture all cotton images on the seedbed. The system workflow is depicted in Fig. 3B. First of all, the operator at the local host remotely connected the IPC of the system through an internet protocol address to gain control. Secondly, the system control software was opened, the automatic image acquisition path point was set, and the automatic image acquisition was started. Then, the IPC and motion control card would control the motion platform to the predefined point and acquire cotton images until all the path points were completed. Finally, the acquired images would be inferred by the trained cotton verticillium wilt model to identify the healthy and diseased leaves and present quantitative results.

### Materials and experiment design

**Fig. 4.**
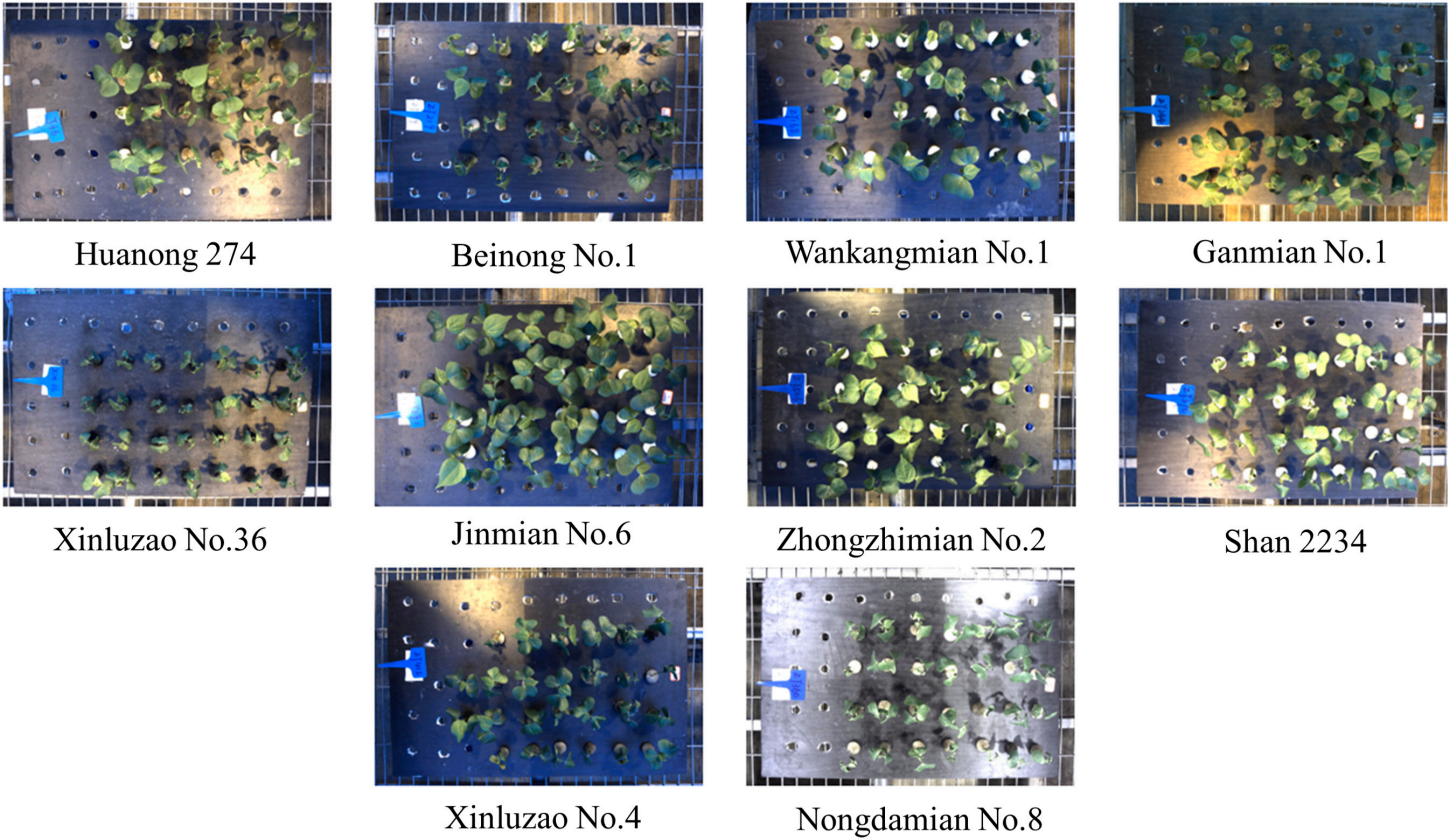
Experimental materials including resistance varieties of Nongdamian No.8, Zhongzhimian No.2, Jinmian No.6, Wankangmian No.1 and Shan2234, and susceptibility varieties of the rest.

According to the cotton verticillium wilt resistance characteristics and natural resistance populations, the 5 cotton varieties with stable disease resistance and the other 5 cotton varieties susceptible to verticillium wilt were used in this experiment, as shown in Fig. 4. The cotton cultivation in this experiment includes the following 3 steps. First of all, put the cotton seeds in vermiculite until seedlings grow. Then, take the cotton seedlings out and place them into a container with culture medium until the lateral roots grow. Finally, inoculate the above seedlings in the spore solution of verticillium wilt pathogen and culture each variety in 2 hydroponic culture pots. The preparation of the above experimental materials took 13 days, and then the images of culture pots were captured on the intelligent observation platform. The image acquisition was conducted at fixed times (1130, 1530, and 1930) every day until the infected leaves withered and fell off completely. The visible light images were automatically collected according to the defined motion locus, which took about 4 min. The system continuously collected cotton images at 64 time points in 22 days, and 2,000 images were selected to build the dataset.

### Model training

Six typical models of 1-stage (RetinaNet, SSD, VFNet, and YOLOF) and 2-stage (Cascade R-CNN and Faster R-CNN) detection algorithms were evaluated in this research. With the captured 2,000 cotton images, LabelImg was applied to annotate the ill and fine leaves. Then, the dataset was divided into a training set and a testing set according to a 9:1 scale. Image augmentation methods were applied during the training, including random flip (in horizontal and vertical directions) and adjustment of color (times 0.5 and 1.5) and brightness (times 0.5 and 1.5) in HSV color space. The hardware parameters for model training were as follows: Intel (R) Core (TM) i9-10900K CPU @ 3.70 GHz processor, 32 GB memory, and GeForce RTX 2080Ti graphics card, while the software environment was based on MMDetection, pytorch 1.6, and python 3.7 in Ubuntu18.04. ResNet50 was used as the feature extraction network (BackBone) of 5 models (Cascade R-CNN, Faster R-CNN, RetinaNet, VFNet, and YOLOF), and VGG16 was used in SSD. Training epoch was set to 24. Batch size was the default value in config files, and a Stochastic Gradient Descent optimizer was applied. Since one graphics processing unit dual-thread training method was applied, learning rate in Stochastic Gradient Descent was set, referring to the rules shown in Goyal et al.’s paper [37].

### Model evaluation

The precision (*P*), recall (*R*), average precision (*AP*), and mean average precision (*mAP*) indexes, computed in Eqs. 1 to 4, were adopted to evaluate the model performance, where the 0.75 intersection over union (IoU) threshold was given. *TP* is the number of true-positive targets, which means the positive targets were correctly identified; *FP* is the number of false-positive targets, which means other category objects were identified as the positive target improperly; and *FN* is the number of false-negative targets, which means the positive targets were identified as other categories by mistake. *P*(*R*) indicates the precision–recall curve, the *AP* value was calculated as the area enclosed by the curve, which could evaluate the detection performance for each class, and the *mAP* is the comprehensive index of model performance for both ill and fine leaf category identification.

$$P=\frac{\mathrm{TP}}{\mathrm{TP}+\mathrm{FP}}\;$$ (1)

$$R=\frac{\mathrm{TP}}{\mathrm{TP}+\mathrm{FN}}\;$$ (2)

$$\mathrm{AP}=\int_{0}^{1}P\left(R\right)\mathrm{dR}$$ (3)

$$\mathrm{mAP}=\frac{{\mathrm{AP}}_{\text{fine}}+{\mathrm{AP}}_{\mathrm{ill}}}{2}$$ (4)

The statistical indicators including root mean square error (RMSE), mean absolute percentage error (MAPE), and *R*-squared (*R*^2^) were adopted for quantitative analysis of regression results, and the formulas are shown in Eqs. 5 to 7, in which ${\hat{y}}_{i}$ is the number of fine or ill leaves by system measurement; *y_i_* represents the number of fine or ill leaves by manual measurement; *N* is the number of samples in the testing set; and $\bar{y}$ is the mean value of $\;{\hat{y}}_{i}$.

$$\text{RMSE}=\sqrt{\frac{1}{N}\sum_{i=1}^{N}{\left({\hat{y}}_{i}-y_{i}\right)}^{2}}$$ (5)

$$\text{MAPE}=\frac{1}{N}\sum_{i=1}^{N}\left|\frac{{\hat{y}}_{i}-y_{i}}{y_{i}}\right|\times 100\%$$ (6)

$$R^{2}=1-\frac{\sum_{i=1}^{N}{\left({\hat{y}}_{i}-y_{i}\right)}^{2}}{\sum_{i=1}^{N}{\left(\bar{y}-y_{i}\right)}^{2}}$$ (7)

Simultaneously, model inference speed was also analyzed by frames per second (FPS), and the formula is shown as Eq. 8, in which *n* is the image number, while *t* is the time consumption for the model inference.

$$\mathrm{FPS}=\frac{n}{t}$$ (8)

### VFNet-Improved

**Fig. 5.**
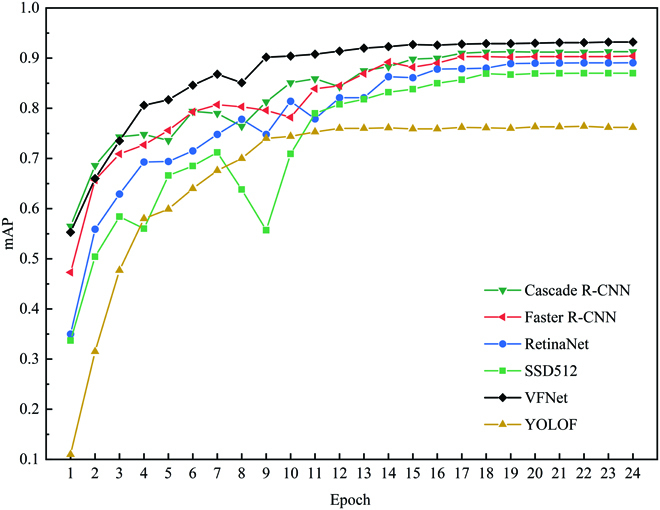
*mAP* of 6 models for different training epochs.

A total of 200 testing images were used for model evaluation. When the IoU threshold was higher, the detection bboxes got closer to the ground truth; therefore, 0.75 IoU threshold was applied. The *mAP* curve of 6 models for different epochs are shown in Fig. 5. After the 16th epoch, the *mAP* of 6 models converged gradually. After converging, the *mAP* of VFNet was superior to other models under the same epoch. The result proved that the VFNet outperformed other models.

Because neither the classification score nor a combination of classification and predicted localization scores could get superior detection performance, in order to rank bounding boxes, VFNet firstly proposed an IoU-Aware Classification Score (IACS) method that simultaneously represented the presence of a certain object class and the localization accuracy of a generated bounding box. Then, a new varifocal loss function was designed to regress the IACS. Meanwhile, the authors used a new star-shaped bounding box feature representation for computing the IACS and refining the bounding box. Finally, a new dense object detector (VFNet) based on the FCOS [38]+ATSS [39] was developed to exploit the advantage of the IACS. Experiments on the MS COCO benchmark showed that VFNet achieved the new state-of-the-art performance among various object detection algorithms. Therefore, the VFNet was adopted for further improvement, and the methods of improvement were as follows.

The traditional geometric transformations were assumed fixed and known. This assumption prevents generalization to new tasks possessing unknown geometric transformations, which were not properly modeled. Besides, the handcrafted design of invariant features and algorithms could be difficult or inflexible for complex transformations. The above 2 drawbacks resulted in difficulty adapting to the cotton leaf shape. Therefore, this study applied Deformable ConvNets v2 (DCNv2) including deformable convolution and deformable region of interest (RoI) pooling in both Backbone and Head of VFNet-Improved to achieve more accurate feature extraction and object detection. A regular grid *R* over the input feature map *x* was used for downsampling. Mathematical expressions of traditional convolution and deformable convolution are shown in Eqs. 9 and 10, respectively, in which *y*(*p*_0_) means each location *p*_0_ on the output feature map *y*, *ω* is weight value, *p_n_* is the enumeration of the locations in *R*, and ∆*p_n_* is the offset corresponding to *p*_0_ on the input feature map *x.* It should be noted that deformable convolution did not learn the offset ∆*p_n_* from the kernel but from each position of the input feature map *x*.

$$y\left(p_{0}\right)=\sum_{p_{n}\mathrm{\epsilon R}}\omega \left(p_{n}\right)\cdot x\left(p_{0}+p_{n}\right)\;$$ (9)

$$\;y\left(p_{0}\right)=\sum_{p_{n}\mathrm{\epsilon R}}\omega \left(p_{n}\right)\cdot x\left(p_{0}+p_{n}+\Delta p_{n}\right)$$ (10)

Mathematical expressions of traditional RoI pooling and deformable RoI pooling are shown in Eqs. 11 and 12, respectively. Given the input feature map *x* and an RoI of size *w* × *h* and top left corner *p*_0_, RoI pooling divides the RoI into *k* × *k* (*k* is a free parameter) bins and outputs a *k* × *k* feature map *y*. *n_ij_* is the number of pixels in the bin and ∆*p_ij_* is the offset.

$$y\left(i,j\right)=\sum_{\mathrm{p\epsilon}\mathrm{bin}\left(i,j\right)}x\left(p_{0}+p\right)/n_{\mathrm{ij}}$$ (11)

$$y\left(i,j\right)=\sum_{\mathrm{p\epsilon}\mathrm{bin}\left(i,j\right)}x\left(p_{0}+p+\Delta p_{\mathrm{ij}}\right)/n_{\mathrm{ij}}$$ (12)

As the offsets ∆*p_n_* and ∆*p_ij_* were typically fractional, bilinear interpolation was implemented at the end of deformable convolution and deformable RoI pooling. The above methods are based on the idea of augmenting the spatial sampling locations in the modules with additional offsets and learning the offsets from the target tasks. Many studies had shown that they were feasible and effective [40,41].

The non-maximum suppression (NMS) algorithm in VFNet calculated the IoU of detection bboxes and set the IoU threshold to remove duplicate boxes of the same object. However, when 2 objects got highly close to each other, the traditional NMS algorithm would remove all the bboxes of the other, resulting in loss of detection bboxes. The soft NMS algorithm was developed based on NMS by improving the confidence reset function through linear or Gaussian weighting. When the detection box score was higher than the IoU threshold, this bbox would be saved instead of directly discarded. Many studies had indicated that soft NMS algorithm could effectively improve the detection accuracy [42]. In this experiment, because the cotton leaves might be very close or even overlapped, the soft NMS algorithm was used to reduce the occurrence of loss of detection bboxes.

**Fig. 6.**
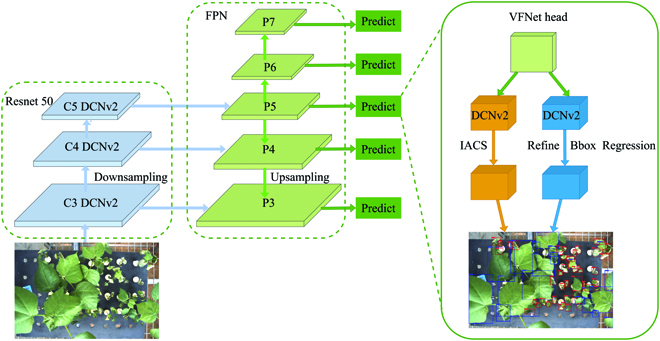
VFNet-Improved architecture for cotton verticillium wilt identification.

Eventually, based on the VFNet detector with DCNv2 and soft NMS, the VFNet-Improved model was applied for cotton verticillium wilt identification, the architecture of which is shown in Fig. 6, and the network of VFNet-Improved was built on the FPN (P3-P7).

### User software design

**Fig. 7.**
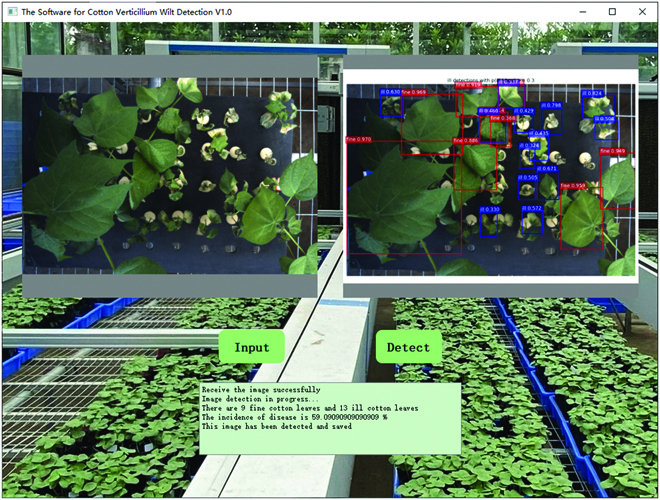
The user software for cotton verticillium wilt recognition.

Automatic cotton verticillium wilt detection software was designed based on the above model and PyQt5, which is shown in Fig. 7. Open the software, then select “Input” button to import the image, and click the “Detect” button to detect the image. The number of “fine” and “ill” cotton leaves and the prevalence rate of the whole pot will be computed, while the detected results will be displayed and saved. The software has packaged all environments and dependencies, which could be conveniently transplanted to other computers. The prevalence rate was computed in Eq. 13, in which *n*_i_ and *n*_f_ were the the number of ill and fine leaves, respectively.

$$r=\frac{n_{i}}{n_{i}+n_{f}}\;$$ (13)

## Results

### Six-model evaluation

The 6 models saved in the last epoch were selected for evaluation, and the results of the 6 models are shown in Table 2: Cascade R-CNN, Faster R-CNN, RetinaNet, SSD, VFNet, and YOLOF had an *mAP* of 0.913, 0.904, 0.890, 0.870, 0.932, and 0.762, respectively, and VFNet outperformed other models. The average detection speed of Cascade R-CNN, Faster R-CNN, RetinaNet, SSD, VFNet, and YOLOF was 11.7, 13.6, 14.2, 46.4, 13.0, and 23.0 images per second (img/s), respectively, and the detection speed of all the models was faster than 10 img/s. The *R*^2^, RMSE, and MAPE of VFNet were 0.999, 0.566, and 0.968%, respectively, for the fine leaf category, which had the best performance. For the ill leaf category, the *R*^2^, RMSE, and MAPE of VFNet were 0.996, 0.831, and 1.436% respectively, and VFNet had the most excellent result.

**Table 2.** *mAP*, FPS, RMSE, MAPE, and *R*^2^ of 6 models in the 24th epoch

Model	BackBone	*mAP*	FPS (img/s)	RMSE		MAPE		*R* ^2^	
				Fine	Ill	Fine	Ill	Fine	Ill
Cascade R-CNN	ResNet50	0.913	11.7	0.933	0.980	1.542%	1.554%	0.998	0.995
Faster R-CNN	ResNet50	0.904	13.6	0.964	1.580	1.825%	2.912%	0.998	0.988
RetinaNet	ResNet50	0.890	14.2	2.179	1.609	5.636%	3.401%	0.996	0.988
SSD	VGG16	0.870	46.4	2.110	2.544	4.372%	5.386%	0.994	0.982
VFNet	ResNet50	0.932	13.0	0.566	0.831	0.968%	1.436%	0.999	0.996
YOLOF	ResNet50	0.762	23.0	2.445	4.502	9.697%	12.253%	0.987	0.919
VFNet-Improved	ResNet50	0.950	10.9	0.557	0.830	0.715%	1.144%	0.999	0.997

### VFNet-Improved model performance

**Fig. 8.**
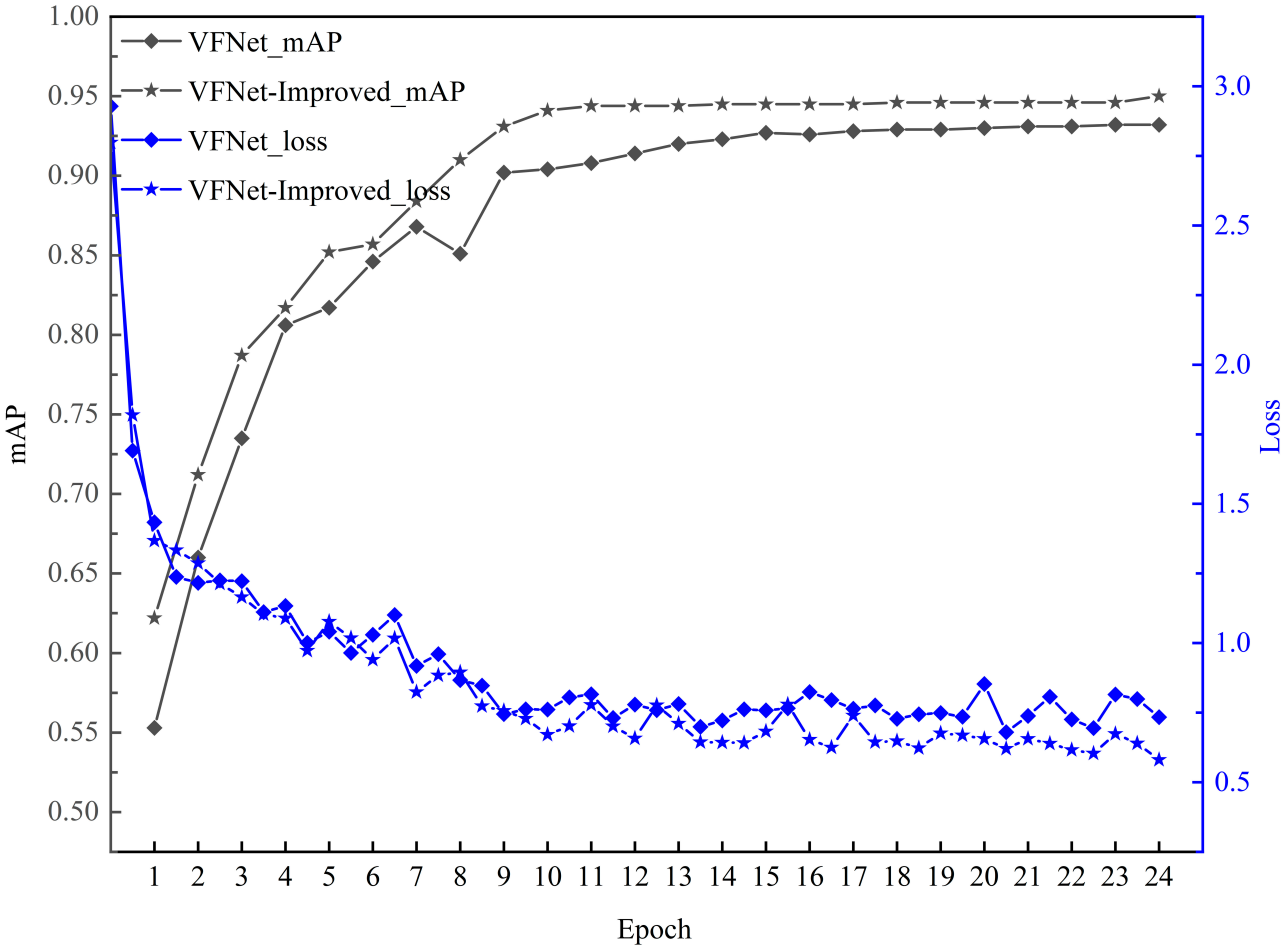
The *mAP* and loss value of VFNet and VFNet-Improved in different training epochs.

**Fig. 9.**
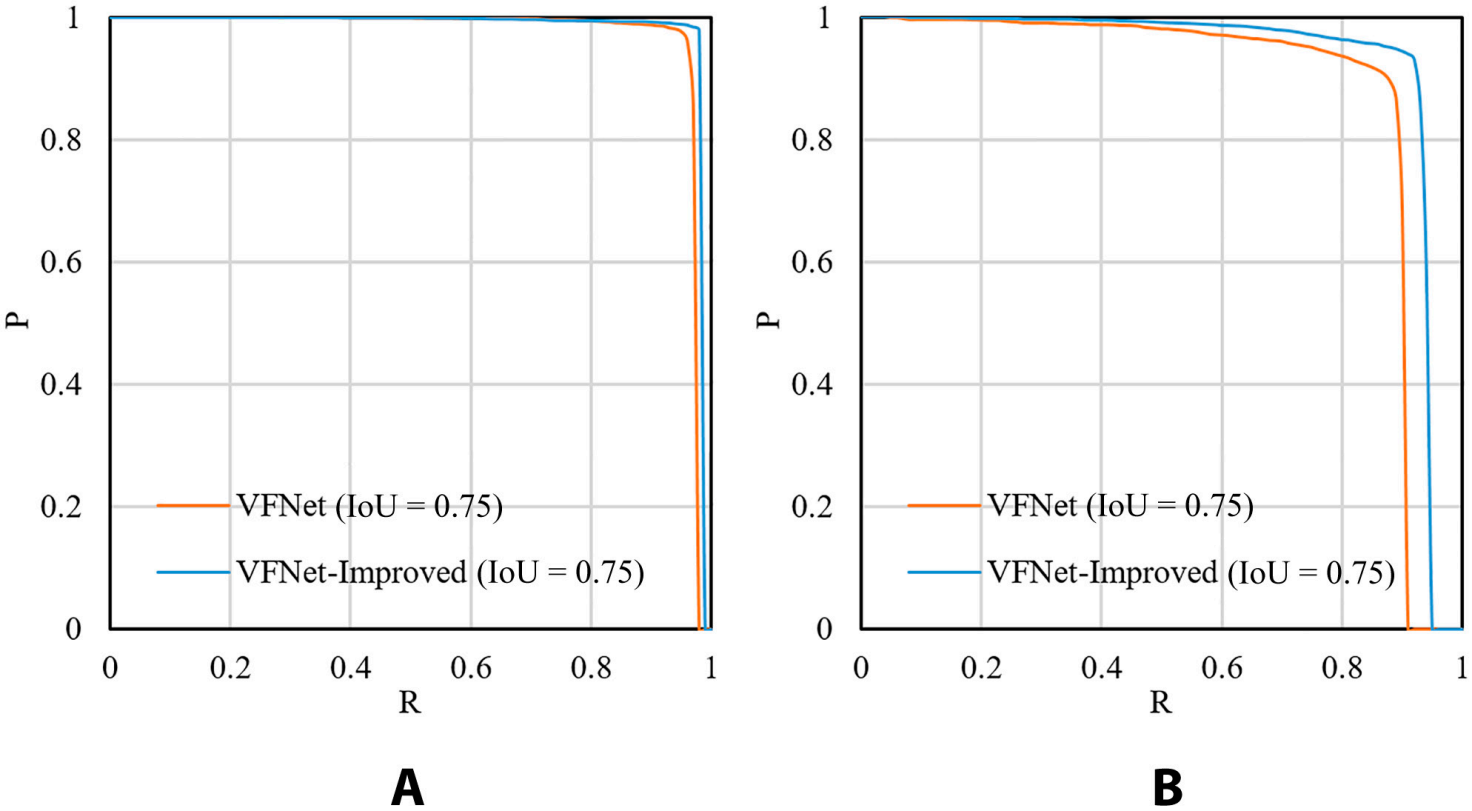
*P*–*R* curves of VFNet and VFNet-Improved for the fine (A) and ill (B) leaf category.

**Fig. 10.**
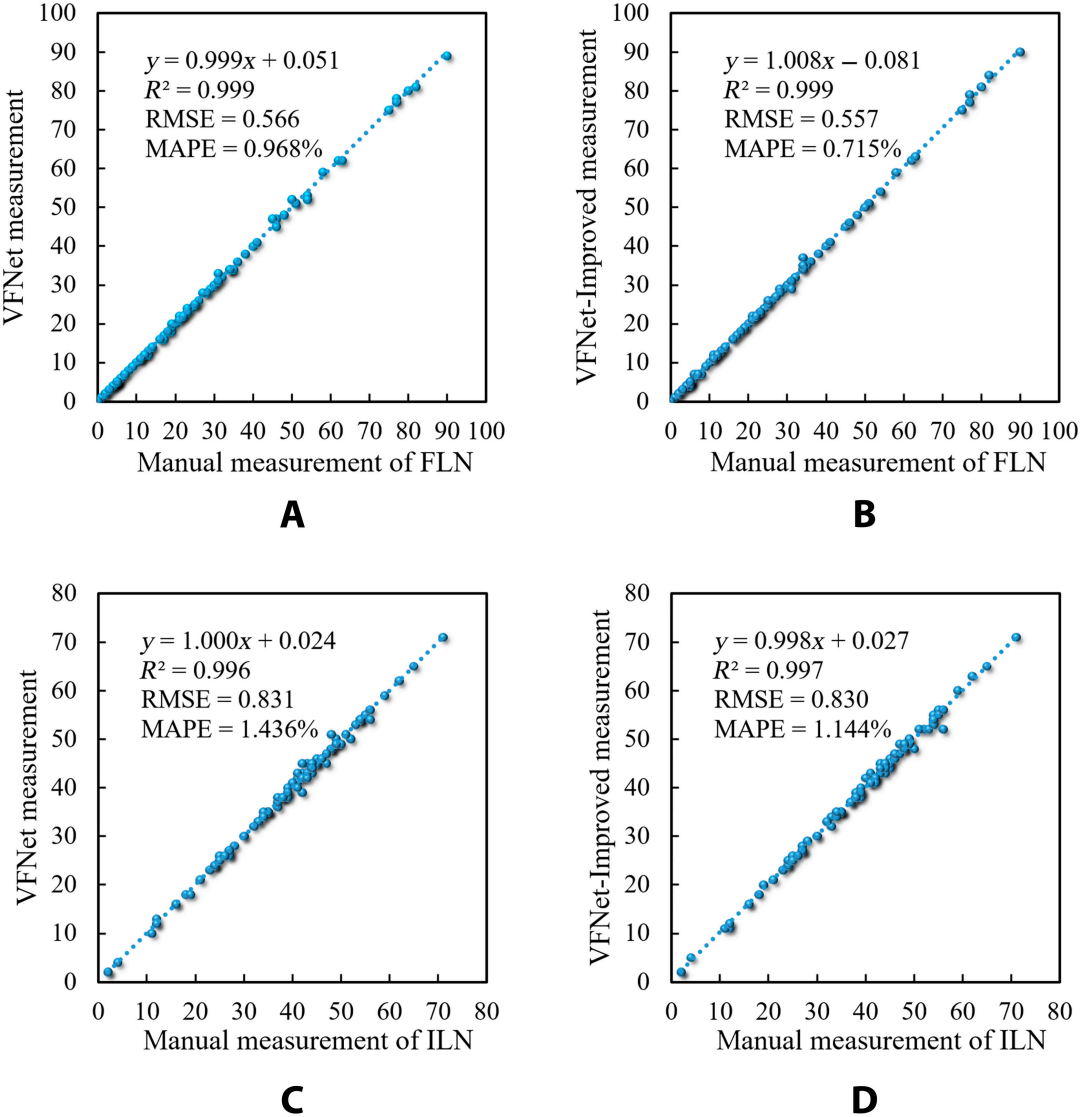
The scatter plots of model measurement versus manual measurement for ILN and FLN.

During different training epochs, the *mAP*, loss value of VFNet, and VFNet-Improved are shown in Fig. 8. The 2 model’s *mAP* converged gradually after the ninth epoch. Obviously, the *mAP* of the VFNet-Improved model had better performance than VFNet. The *mAP* of the VFNet-Improved model improved by 1.8% compared with VFNet in the 24th epoch. The loss value of VFNet-Improved was also lower than that of VFNet. *P*–*R* curves of ill and fine leaf categories are shown in Fig. 9A and B, respectively, where 0.75 IoU thresholds were chosen for evaluation. In *P*–*R* curves, a larger area (AP value) between the curve and the positive direction of *X* and *Y* axes indicated a better detection effect. The results showed that the detection results obtained by VFNet-Improved were superior to those of VFNet, and VFNet-Improved had a better effect on the ill leaf category than fine leaf. The scatter plots of model measurement versus manual measurement for ill leaf number (ILN) and fine leaf number (FLN) are shown in Fig. 10. For the fine leaf category, the RMSE, MAPE, and *R*^2^ of VFNet-Improved were 0.557, 0.715%, and 0.999, while those of VFNet were 0.566, 0.968%, and 0.999, respectively. Meanwhile, for the ill leaf category, the RMSE*,* MAPE, and *R*^2^ of VFNet-Improved were 0.830, 1.144%, and 0.997, while those of VFNet were 0.831, 1.436%, and 0.996, respectively. The results showed that the VFNet-Improved outperformed the VFNet model, especially for the ill leaf category.

### Dynamic observation of cotton verticillium wilt

After comprehensive comparison and detailed analysis of the above results, the VFNet-Improved model was finally adopted to the user software to dynamically observe cotton verticillium wilt. Meanwhile, we also designed a validation group to test the system performance. According to the resistance and susceptible characteristics of verticillium wilt described in “Chinese Cotton Varieties and Genealogy”, 3 resistant varieties Wankangmian No.9, Xinluzao No.30, and Zhongzhimian No.2 and 3 susceptible varieties (Daizimian No.16, Xinluzao No.4, and Xinluzao No.36) were selected to participate in testing. The first day of the experiment was the time when cottons were inoculated with *Verticillium dahliae*. The images taken on the 9th to 21st day (observation period) were tested by the VFNet-Improved model. After analysis of the detection results, the prevalence rates of 6 cotton varieties during the observation period were obtained and then the curves were drawn respectively.

**Fig. 11.**
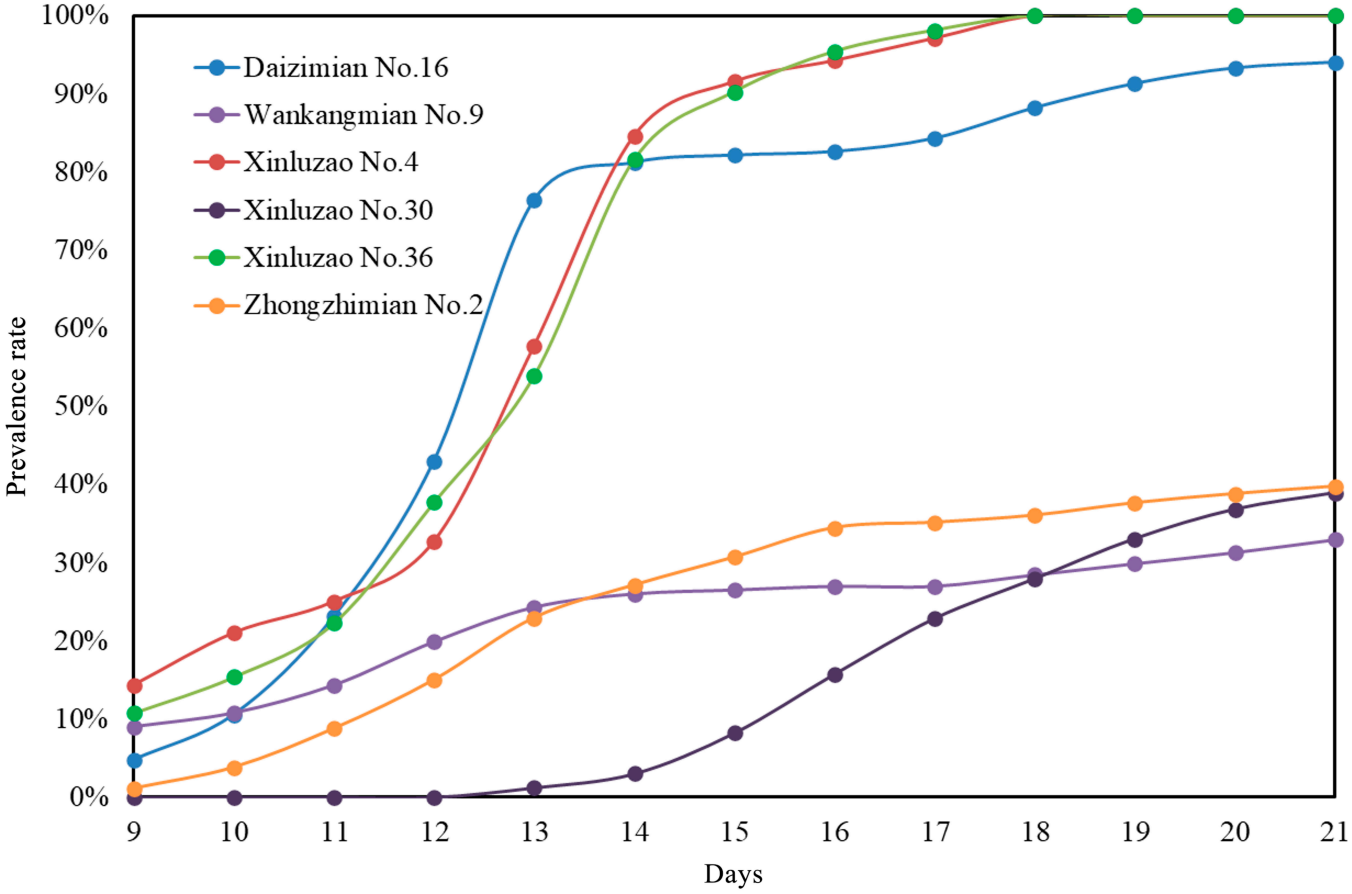
The prevalence trend of cotton verticillium wilt for 6 cotton varieties.

**Fig. 12.**
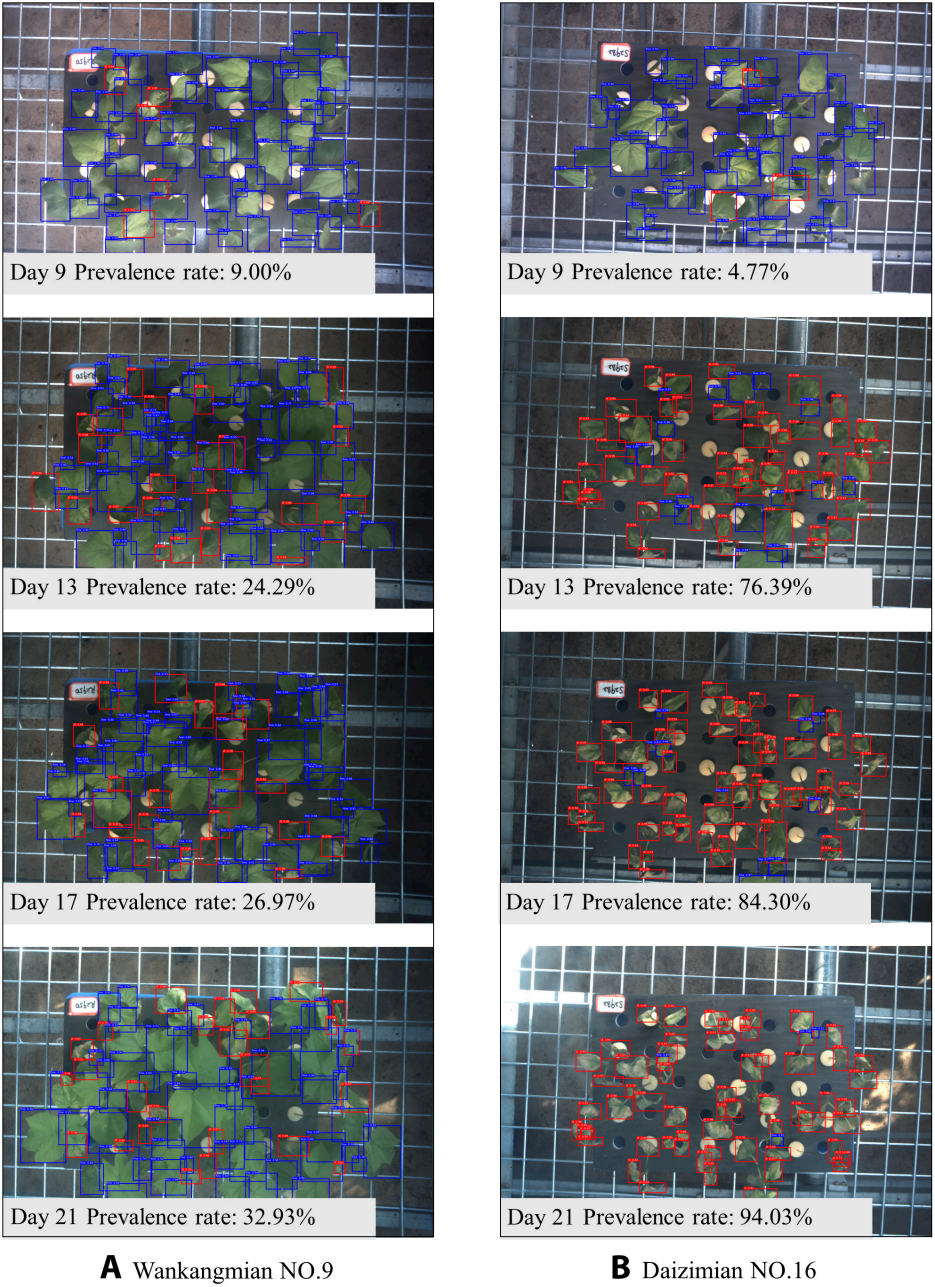
Dynamic observation comparison of cotton verticillium wilt for resistant (A) and susceptible (B) varieties.

As shown in Fig. 11, on the ninth day, the prevalence rates of all varieties were lower than 20%. The prevalence rates of 3 susceptible varieties accelerated from the 10th day. After the 16th day, the prevalence rates were all more than 80%, and then increased slowly until the leaves were completely withered. The prevalence rates of resistant varieties were lower than 20% on days 9 to 12 and increased slowly from the 12th day to the last day. The final prevalence rates were all lower than 40%. Meanwhile, the image detection results of Wankangmian No.9 and Daizimian No.16 were also selected to make a time sequence diagram, and the detection images are shown in Fig. 12. The experimental results proved that the system could provide an efficient and reliable tool for dynamic observation of cotton verticillium wilt.

## Discussion

The verticillium wilt is the main disease in cotton production. However, the traditional investigation method in cotton breeding and genetic research is still manual, which is inefficient, labor-intensive, and subjective. Besides, most research is in the field, in which the weather is complex, unpredictable, and disturbing. Therefore, a platform based on greenhouse is necessary. The greenhouse platform could collect and analyze the dynamic growth phenotyping information of cotton under a controlled environment. Combined with environmental factors, genomics, and phenology data, the system could help achieve intelligent and efficient breeding. Meanwhile, most high-throughput phenotypic platforms based on greenhouse around the world were “plant to sensor”, in which plants were grown on a conveyor belt and transported to an imaging sensor [18]. However, because cotton seedlings were slender, the movement would cause damage and position change. Therefore, in order to achieve dynamic and undamaged observation of cotton verticillium wilt, this research designed an intelligent vision-based system with the mode of “sensor to plant”. With the high-precision motion control platform, unmanned image acquisition was carried out by a predefined motion locus, and it took only 4 min to automatically capture all cotton images on the seedbed, which was efficient, reliable, and flexible. This novel intelligent system based on the platform was able to accurately investigate cotton verticillium wilt and quantify the prevalence rate with high throughput and provided an efficient and reliable method for further dynamic observation of cotton verticillium wilt.

**Fig. 13.**
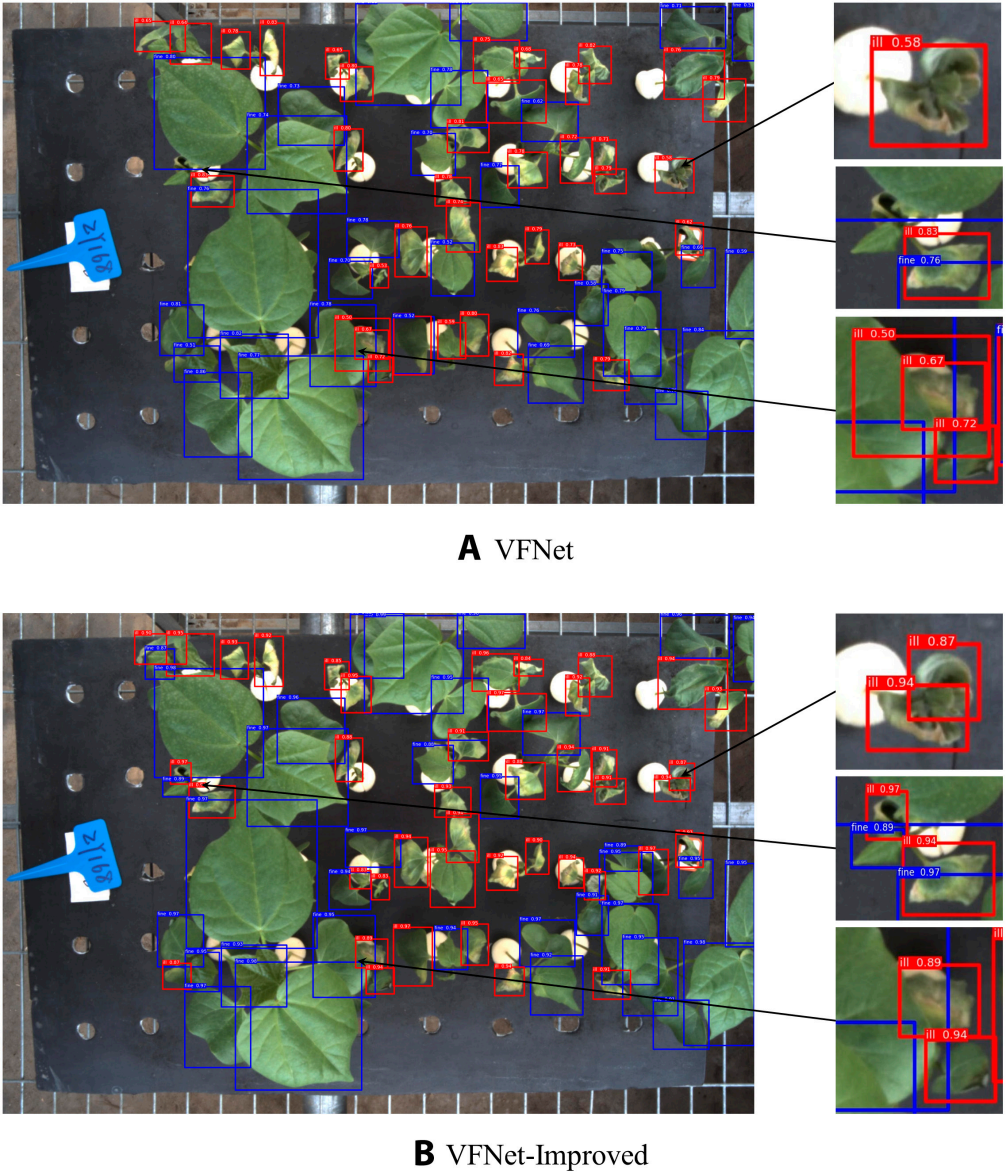
Detection comparison between the VFNet (A) and the VFNet-Improved (B) model.

Additionally, the cotton verticillium wilt recognition software has been developed based on VFNet-Improved. Because of the multiscale characteristic and shelter between cotton leaves, which would result in low detection accuracy and inaccurate bboxes, specific identification algorithms for cotton verticillium wilt should be developed. After comparing with SSD, RetinaNet, YOLOF, Faster R-CNN, and Cascade R-CNN, the VFNet model proved to be more effective, which proposed a new star-shaped bbox feature representation for IACS prediction and bbox refinement. Based on the VFNet, the optimal methods including DCNv2 and soft NMS were adopted. The DCNv2 would make the position of input feature map *x* expand dynamically, which could achieve more accurate feature extraction during training. The soft NMS algorithm would improve the detection effect during inference by confidence reset function [45]. Eventually, the *mAP* of VFNet-Improved using deformable convolution, deformable RoI pooling, and soft NMS methods improved by 1.8% through evaluation. For details of the improvement, detection effects of VFNet and VFNet-Improved are shown in Fig. 13A and B. VFNet would result in missed or wrong detection in the case of leaf occlusion and small target as shown in Fig. 13A, while this situation was greatly reduced by VFNet-Improved as shown in Fig. 13B. That is to say, instances of missed or wrong detection were reduced by DCNv2 and soft NMS, and the detection accuracy could be effectively improved eventually. The FPN was adopted to obtain the feature maps with different scales, which could help promote the recognition performance for different leaf size, and soft NMS was applied to optimize the confidence function, which could help improve the recognition accuracy for partial leaf occlusion. However, if the leaves were completely occluded, the occluded leaves would not be viewed and recognized, which could only be solved by adding a multiview camera. Besides, since the deep learning algorithm was data-driven, we have collected images at different time points in the training dataset and performed image enhancement to mitigate the influence of light change and to enhance the robustness and generalization ability of the model. In conclusion, the VFNet-improved model had a good performance during the verticillium wilt investigation and provided an efficient, accurate, and reliable tool for this system to observe cotton verticillium wilt dynamically.

## Conclusion

The verticillium wilt is one of the most critical cotton diseases, which is related to cotton production. This study has demonstrated a novel intelligent system for dynamic observation of cotton verticillium wilt on the seedbed, which would provide a feasible and effective tool for cotton breeding and disease resistance research. The main points of the research were as follows:

1) Firstly, an intelligent vision-based system has been developed to dynamically observe cotton verticillium wilt, in which the “sensor to plant” mode and high-precision motion have been implemented. The system would take only 4 min to automatically capture all cotton images on the seedbed, which was efficient, reliable, and flexible.
2) Secondly, as to the cotton verticillium wilt identification, the research proved that the VFNet outperformed the other object detection network, and the optimized VFNet with deformable convolution, deformable RoI pooling, and soft NMS could achieve a *mAP* of 0.950, which had extremely high consistency with manual measurements. Moreover, the MAPE for FLN and ILN measurement of the VFNet-Improved model was reduced by 25.91% and 20.33%, respectively, compared with VFNet, which was innovative and meaningful for the cotton verticillium wilt identification.
3) Thirdly, the dynamic observation results proved that this system was able to investigate cotton verticillium wilt with high accuracy and efficiency, and quantify the prevalence rate of different varieties at different stages, which could help achieve intelligent and efficient breeding.

In the future, we plan to carry out grading research on cotton verticillium wilt, accurate recognition of cotton verticillium wilt in the early stage, and correlation analysis between phenotype, environment, and gene data.

## Data Availability

The cotton images and measured data in this study are available upon request by contacting the first or corresponding authors.

## Supplementary Materials

Supplementary Materials Video S1. The video of system application.
